# Interviews with Global Pharmacists and Healthcare Professionals in Great Britain to Establish Personal Experiences around Professional Development Activity

**DOI:** 10.3390/pharmacy10010007

**Published:** 2022-01-01

**Authors:** Ricarda Micallef, Reem Kayyali

**Affiliations:** Department of Pharmacy, Kingston University London, London KT1 2EE, UK; r.kayyali@kingston.ac.uk

**Keywords:** pharmacist, healthcare professional, professional development, CPD, CE, global

## Abstract

Professional development activity is needed to ensure practitioners are up to date and providing optimal patient care. This includes, but is not restricted to, mandatory continuing professional development (CPD) or continuing education (CE) requirements, which differ by professions globally and within countries. This study aimed to investigate perceptions, participation, and individual practice for healthcare professionals in Great Britain (GB) and pharmacists globally to identify similarities and differences after the introduction of revalidation for pharmacists in GB. Qualitative data was received through interviews, which was analysed using content analysis. In total, 24 interviews were completed with pharmacists registered globally, and healthcare professionals registered in GB. A culture of CPD was seen for healthcare professionals in GB and globally for pharmacists; there was no consistent model. Face-to-face activity was common, with an increase in online provision, especially where large geographies were seen. Most learning was completed in the professional’s own time. Multiple providers were seen, with the evaluation of events using questionnaires being commonplace. Different formats of learning were useful for different topics, with skills learning being better when face-to-face. Although varied requirements were in place, regulation should support patient-based practice outcomes. This study showed that commitment to learning was similar in different professions in GB and by pharmacists globally, with similar benefits and challenges.

## 1. Introduction

Professional development activity is needed to ensure practitioners are up to date with current drugs and guidelines, as well as to ensure that they are providing optimal patient care. Continuing Professional Development (CPD) is a lifetime commitment of providing care in a safe and effective way [1]. Continuing Education (CE), or CPD as professional development activities are commonplace for healthcare professionals globally, although the mandatory requirements in place are currently varied. Professional development activity is essential for pharmacists to ensure their knowledge and competence is maintained throughout their career; however, learning is not restricted to only mandatory requirements, as it is also self-driven. Whilst CE has a primary focus on pure participation in activity and recording of the hours of activity completed, CPD demonstrates reflection on learning needs, planning, participation, and evaluation. It has been noted that CPD offers a greater return of investment compared to CE, as there is a greater focus on context and application [2,3]. It has been noted that CPD must facilitate changes in behaviour to support advancement of pharmacy practice [4].

For pharmacists, multiple reports have reviewed the requirements set up at a global level for mandatory professional development activity [1,5,6,7,8] and these show a wide range of variation amongst countries. In the International Pharmaceutical Federation (FIP) global report on CE/CPD in 2014, it was seen that 33 out of 66 surveyed countries had no requirements in place for pharmacists. Where a mandatory requirement to demonstrate CE or CPD is seen, the requirements are wide and varied across and within countries. Current requirements may be linked to the length of time that the profession has been established, the role of the pharmacist, or current regulation requirements [5]. Driesen et al. [9] noted that there is no global model in place for professional development activities of pharmacists.

From looking at the international pharmacist CPD reports available [1,5,6], mandatory CPD systems are in place in Australia, Canada, Great Britain (GB), Ireland, Malaysia, Namibia, New Zealand, Northern Ireland, Oman, Portugal, Singapore, and the United Arab Emirates (UAE). It is noted that pharmacy in Portugal and the UAE is regulated by Government/Ministry, and these have CPD credit systems, akin to CE due to hours of training that has been completed being recorded, whereas all other listed countries have mandatory CPD for pharmacists that is regulated by councils or boards at the individual state level in a country, requiring reflection of practice. The United States of America (USA) is the only country listed in the reports, which looked at data until 2014, as having a mandatory CE system, in some states. Since 2014, CPD is now much more commonplace in the USA [10].

Models or formats used for CE/CPD differ both globally and within countries, with differences seen for models and preferences for learning [11]. Currently providers of professional development activity have no global reference for requirements [12] due to differing models and expectations. Where there are no CE/CPD requirements in a country, pharmacists may still want to engage in learning activities, so identifying current approaches used may benefit those introducing models in the future. In GB, as in countries globally, healthcare professions are registered by regulatory bodies, along with the majority having professional bodies to support them. Nurses and doctors are the first and second largest healthcare professions in GB, with pharmacists being the third largest and dentists being the fourth largest healthcare profession [13]. The Nursing and Midwifery Council (NMC) is the regulator for nurses and midwifes [14]. The Royal College of Nursing (RCN) is the professional body for nurses. The General Medical Council (GMC) regulates the medical profession. The British Medical Association (BMA) is the trade union and professional body for medics, providing events and learning materials, including access to the British Medical Journal (BMJ) and BMJ learning, an online learning platform. The regulator for pharmacy in GB is the General Pharmaceutical Council (GPhC). All GPhC registrants have access to learning by the Centre for Pharmacy Postgraduate Education (CPPE). The professional body for pharmacists is the Royal Pharmaceutical Society (RPS). The General Dental council (GDC) regulate dentists, and the health and care professions council (HCPC) regulate 16 professions working in health, psychological, and social work professions including radiographers, paramedics, physiotherapists, and occupational therapists [15]. Health Education England (HEE) provides an overview of workforce planning and provides education to those in health professions [16].

In 2014, Tran et al. [1] published information about CPD requirements in various countries, comparing that of dentists, nurses, pharmacists, and physicians. Tran’s study used data for GB that had been gathered in 2013 through a literature review. Since the review by Tran in 2013, [1] two professions in GB have seen changes in the regulatory requirements of CPD; pharmacists and dentists. In 2018, revalidation was introduced for pharmacists in GB, incorporating CPD, which updated regulatory requirements for re-registration. Revalidation is in place to support public confidence in professionals [17,18]. Prior to the introduction of revalidation for pharmacists, there was a requirement for nine CPD cycles to be completed annually. With revalidation, this has been amended to be four CPD cycles, but also to complete a reflective account and carry out a peer discussion. For dentists, as of 1 January 2018, there is an enhanced CPD process [19] replacing the old process, which has been in place since 2008, and all dentists will transition to the new scheme at the end of their five-year CPD cycle. 

As with CPD requirements, revalidation requirements are set by each professional regulator. Whilst both professions with updated requirements have seen a reduction in overall requirements, there is more emphasis on verified learning and showing the impact of learning on practice. A review of CPD requirements of UK health professionals published in 2020 acknowledged that the regulatory requirements are the minimum expectation and professional development activity is likely to exceed that set by the regulator [20].

Whilst previous studies have looked at requirements and comparisons of CPD/CE globally for pharmacists, or compared requirements for GB based healthcare professionals, no qualitative studies can be found. This is the first in-depth qualitative analysis, including interviews from practising professionals, and representatives of professional groups, focusing on experiences of professional development activity for healthcare professionals in GB and pharmacists globally. As GB based pharmacists have amended CPD and revalidation requirements in place, a comparison of other healthcare professionals in GB, plus global pharmacists, allows an understanding of how GB pharmacists requirements compare in terms of local healthcare colleagues, but also global pharmacy colleagues. With increased emphasis on a multi-professional approach to patient care being seen, perceptions from learning providers and professional bodies will also support understanding of alignment in practice, to identify any learnings that can be shared across professions, both in GB and globally. 

The aim of this study was to investigate perceptions, participation, and individual practice for regulatory professional development activity requirements of healthcare professionals and healthcare regulators or support bodies in GB and pharmacists globally, in terms of provision, uptake, and attitudes, in order to identify similarities and differences after the introduction of revalidation for pharmacists in GB. 

## 2. Materials and Methods

### 2.1. Study Design

Qualitative data from healthcare professionals in GB plus pharmacists globally was received using semi-structured, one-on-one interviews. This approach was taken to gain perceptions on how regulations or practice can affect individual practices. An interview proforma consisting of 13 questions was designed. It aimed to understand current provision, support, participation, and evaluation of professional development education and training activities. In addition, it captured thoughts about the best options and attitudes towards preferences for delivery of learning events, to compare and contrast current practice and views. The interview schedule received face validation for content from three pharmacists and a nurse in GB not involved in the study. A copy of the interview schedule can be found in Appendix A. To ensure study integrity in the design and analysis, the COREQ checklist was used. This can be found in Appendix B.

### 2.2. Participants: Sampling and Recruitment

Human participants were involved in data collection through semi-structured interviews, completed either face-to-face or via telephone, when face-to-face was not possible. The lead author completed all the interviews. 

Potential participants were contacted by email and purposive convenience sampling was used through local contacts to gain participants. Contacts in pharmacy, nursing, medicine, and dentistry from previous committee work were emailed to gain an understanding of requirements and experiences for colleagues in other pharmacists in GB. Only one individual from each profession, country, or organisation was targeted to gain a perspective of how individual practice is interpreted, noting that perceptions would be self-reported. Pharmacists from countries including Great Britain as the home country, as well as Ireland, Australia, Belgium, Canada, Malta, the USA, and New Zealand were approached as colleagues who had an interest in professional development activity, due to previous conference attendance. Colleagues registered in other countries, along with those registered on the Overseas Pharmacists’ Assessment Programme (OSPAP) course in GB were also approached. OSPAP is a course for pharmacists from outside of the European Union who wish to join the register in GB. Practising pharmacists were chosen for the interview to understand the reality of how requirements set by regulators are applied in practice. Participants, when contacted, were made aware of the study contributing towards the lead researcher’s PhD and were made aware of her previous research and practice as a pharmacist.

### 2.3. Data Collection

Individuals who agreed to participate in an interview were emailed an information sheet, outlining the study aims and objectives, as well as the background of the researchers, including the right to withdraw from the study and a consent form, which they were asked to read, sign, and return prior to the agreed interview time if not available face-to-face. Those who were being interviewed face-to-face were given a copy of the information sheet and consent form prior to the interview commencing. Where face-to-face interviews occurred, the researcher travelled to a convenient workplace location for the participant. All other interviews occurred over the telephone. Verbal or written consent was obtained for recording, as appropriate. Interviews lasted between 10 to 15 min. All interviews were voice recorded and transcribed verbatim prior to deletion. No other notes were made during the interviews. The interviews took place between February 2017 and October 2018. All those who initially agreed to be interviewed completed an interview, in order that all views and experiences were recorded, and to ensure representation of countries and professions in GB. One female member of the research team (RM) who was completing her PhD at the time of the study and had three years prior experience of qualitative research completed all the interviews and completed the transcriptions. No other individuals besides the researcher and participant were present during all interviews. 

### 2.4. Data Analysis

Inductive content analysis was used to combine data gathered through interview to give a general statement. Working with qualitative data, content analysis allows the integrity of the narrative to be maintained, using a summary along with supporting excerpts [21]. The questions were used as codes to gain responses for comparison across professions and countries. Frequency counts were used as a form of content analysis to highlight the most common topics or words given during answers [22]. This was completed by one member of the research team (RM) with the other member (RK) reviewing all transcripts to ensure the accuracy of findings. Analysis was completed manually. As the aim of the study was to identify perceptions in approach about current practice, the directed content analysis approach allowed for validation of current knowledge and could determine relationships between codes. Transcripts were read to ensure there were no transcription errors, and then read again to enable immersion. Quotes were used to highlight any key messages highlighted from the coding. 

## 3. Results

Interviews took place with one representative from each of the following healthcare practitioners in GB (*n* = 5): dentist, hospital doctor, nurse, paramedic, and radiographer. Interviews took place with one member of personnel from the following support/training bodies in GB (*n* = 5): BMA, BMJ learning, CPPE, HEE, and RPS. There were 14 pharmacists interviewed who practiced in different countries. One pharmacist was registered in each of Australia, Belgium, Chile, GB, India, Iraq, Ireland, Malaysia, Malta, Pakistan, Philippines, and USA that was interviewed. In addition, two pharmacists from New Zealand were interviewed. Despite multiple emails, no representative from South Africa or Canada was recruited. Where quotes are used in the text, the role of the GB based healthcare professional, or the country of the global pharmacist, are given. As a GB pharmacist was interviewed, this can be used as a comparison for both professions in GB and pharmacists globally. There is a mixture of CPD and CE requirements around the world, along with countries who do not have any regulatory requirements. Interview findings have been summarised in Table 1 and Table 2.

### 3.1. Professional Development Activity Practices

In GB, a culture of CPD is seen with all professions that were interviewed engaging in CPD (*n* = 5/5), echoing GB pharmacists. Of these, hours of CPD are specified for nurses and dentists, with the HCPC having a portfolio approach to learning. For pharmacists, the number of cycles that need to be completed is specified. For doctors, nurses and pharmacists, CPD requirements are undertaken as part of mandatory revalidation activity. Of the 14 pharmacists that were interviewed, 7 were from countries where they needed to undertake CPD: Australia, GB, Iraq, Ireland, Malaysia, and New Zealand (2 pharmacists); 3 pharmacists had to undertake CE: Belgium, Philippines, and the USA; and the remainder had no requirement. Of the nine who engaged in CPD/CE, six of these were a points-based or credit-based system (Australia, Belgium, Malaysia, Philippines, New Zealand, and the USA). Hours of CPD (Australia, New Zealand) or CE (Belgium) were also required in some cases to gain credits along with a specification of the number of CPD cycles to complete (GB). It must be noted that the interview responses represent individual practising pharmacists and the requirement on their practice, e.g., in the USA the pharmacist interviewed had a CE requirement, whereas other pharmacists from other states may be subject to CPD requirements.

### 3.2. How Is Learning Achieved and Verification of Learning

Most professions in GB could achieve their learning through multiple formats. The paramedic in GB was the only professional who stated learning is mainly through attendance, as seen in the quote below.

*‘A lot of it is attendance at courses and sort of higher education things then you have to write up how it applies to what you are going to do.’*

Paramedic.

All other professions noted a mixture of available activities, with opportunity to undertake face-to-face or alternative methods of learning. Providers spoke about their online training provision (BMJ learning) or portfolio of learning options (BMA, CPPE). Face-to-face learning activity was the most common seen from the interviews, although in Western countries, there was an increase of online courses available, with online provision noted in 7 out of 13 countries for pharmacists (Australia, Belgium, GB, Ireland, Malaysia, New Zealand, and the USA,) allowing for flexibility of learning. Where online learning was mentioned, this was always in conjunction with a face-to-face offering. Face-to-face activity comprised multiple activities, including conferences, postgraduate programmes, or courses. The opportunity to not just undertake face-to-face, but also participate in alternative options was noted as positive:

*‘We have a lot of formats available. We have online learning, so we have a number of e-learning courses… Face-to-face workshops.’*

CPPE.

Gaining the credits can be complex to understand, with Australia, Belgium, and New Zealand having scaling systems in place. The groups of activity seen in Australia and New Zealand are similar. The quote below outlines the requirements in New Zealand:

*‘Every year you have to record your CPD and it is done online. And you have to get 20 points a year minimum, but over 3 years you have to get 90 points. And then they split it up and you need a certain number of group one points, a certain number of group two points and a certain number of group three points. Now, group one points–that just means like, reading an article or, reading a paper or something like that, so you can show you have done something. In group two though, you have to do learning, but also be assessed on it, so there will be, if you go to a conference and they give you a test at the end and you pass it, you can put that down. And then group three you need a learning partner and you have to do, it is a combination of doing all of your group one and group two. So you are learning and then showing how you are using that in practice. So an example could be like, um, one of the group three points ones I did was writing a community acquired pneumonia guidelines. You do the research then you write the guideline, then you put it in practice and you see how practice is changing as a result.’*

New Zealand 2.

Ireland has a separate organisation in place to implement a CPD system, and supports pharmacists to achieve requirements, and they also commission education and training programmes. This is outlined in the quote below:

*‘So every year, a pharmacist needs to apply for continued registration and then they also need to engage with the Institute of Pharmacy (IIOP)…they need to maintain an e-portfolio on the IIOP website… They need to demonstrate they are engaging in CPD… we must be able to have a direct evaluation of a pharmacists’ knowledge, skills, and competencies in a range of patient facing roles… we have copied the Ontario model, so a pharmacist will come to a central location. It is run twice a year, on Saturday and Sunday each weekend, and they go through eight what we call standardised patient interactions…then they move to a clinical knowledge review, which is an online e-assessment and they sit at the computer with, I think, 16 cases, each has 3 multiple choice questions on each, it is open book, and they need to complete that in just under 2 h…’*

Ireland.

Interestingly, although no formal requirement exists in many countries, courses are still offered, often with high participation rates, as seen in Malta, as an example below.

*‘There are no legal requirements but there are continuing education courses routinely. I would say with a very high participation of over 60%, 70% of pharmacists, even though it is voluntary.’*

Malta.

Certification or proof of attendance/participation is commonplace to fulfil CPD requirements. Certificates are required for three professions (nurses, doctors, and dentists) for verifiable CPD. These are required for submission to the regulator for audit or for use during an appraisal for doctors. However, all professions stated that the regulator only looks at a small sample of records submitted. Of the countries engaging in CPD/CE, certification or proof of attendance/participation is commonplace in all but Iraq. Even where CPD/CE is not a requirement, it is seen that certificates are issued at times, such as in India. 

### 3.3. How and When Learning Takes Place

A large culture of carrying out learning in the professional’s own time is seen both in GB and globally. All professions in GB stated that some learning should be completed independently (*n* = 6/6). Doctors are seen to be given protected time during work hours, and dependent on the hospital trust, nurses may be given this too. Protected time for pharmacists is seen in hospitals in New Zealand and the USA, but not in the community sector. Australia and Belgium do show that some money could be claimed back from attendance. Evenings are a very common time for activity to take place (dentist, BMA, CPPE). Some activity predominantly occurs during the day (doctor) or mainly in the evenings (dentists), with other professions having flexibility of events during the day or the evening (nurses, paramedics, pharmacist). Radiographers noted weekend events being common. The flexibility of offering learning events both during the day, evenings, and weekends is widely seen globally for pharmacists. Where distance learning is offered, this can be always accessed. Geography does play a part in the time of activity, as travelling time to locations and the associated cost can affect attendance numbers, as seen in the quote below.

*‘And making the time to go after work is very difficult, and travelling, you don’t know if it is local to you. And cost of travelling can be a bit much.’*

Dentist.

Small countries, such as Malta, do not face problems with attendance. In the USA, there are more daytime learning activities held, as well as in Iraq due to political reasons as seen in the quote below.

*‘During the day and sometimes the weekend…because of the situation it has moved away from night and into more of the daytime.’*

Iraq.

### 3.4. Providers of Professional Development Activity

Professional bodies are the predominant providers of training across the board, although employers, universities, and pharmaceutical companies also provide learning opportunities. Globally for pharmacists, there are a few country-wide specific providers e.g., Instituut voor Permanente Sudie voor Apothekers (IPSA), translated as the Institute for Continuing Education for Pharmacists, for Flemish speakers and Brussels based pharmacists, along with *Société Scientifique des Pharmacies Francophones* (*SSPF*) for French speaking pharmacists, in Belgium, CPPE in GB. Countries including Ireland and USA have accrediting organisations to support commissioning and accreditation of training for pharmacists, with Ireland using the Irish Institute of Pharmacy (IIOP) and USA having the accreditation council for pharmacy education (ACPE). The ACPE accredits provider organisations. 

### 3.5. Follow-Up after Events

A variety of support material is seen to support the application of learning into practice. These include case studies being used during the session (nurse, dentist, CPPE, Chile, Malta), vignettes (nurse), online material (CPPE, doctors, New Zealand), handouts (doctors, pharmacists in GB, Iraq, USA), CPD support tools providing reflection points from the session (pharmacist, radiographer, Ireland), and post course assessments (CPPE, New Zealand, Australia, Belgium). Support targeted depending on the content of the session was mentioned, as seen in the quote below:

*‘Well, they will be your sort of conventional resources which are your written material… You could have a memory stick with information on it, you could have access to online material with a special code you could have. You could have leaflets, pamphlets, and material. It is a combination. You could sometimes have access to MCQ questions and interviews online, all sorts of things like that. It is targeted.’*

New Zealand 1.

The BMA send out a list of the top information to remember after the event with links to further learning. In some cases, it was stated that no support is given (paramedic). Pharmacists in Belgium send out a list of the top information to remember after the event with links to further learning. In some cases, it was stated that no support is given (India, Philippines). When asked about any tools that are not currently utilised that may be of benefit, review, and evaluation of learning, critical analysis/appraisal and handouts were mentioned.

Evaluation of events takes place in numerous ways including online follow-up survey (nurse, BMA, Ireland, USA), self-reflection (radiographer), and the most common, evaluation form (paramedic, pharmacist, CPPE, Australia, Belgium, pharmacist in GB, Iraq, Malta, New Zealand, Philippines). It is noted, however, that this may not be seen as true evaluation, as seen below:

*‘We have feedback forms that everybody does. But that’s probably not true evaluation because if you were going to do proper evaluation you are looking at what difference has that made to one’s practice which you can’t measure in a two-hour workshop. So, at the end of a two-hour workshop you can only measure at best I think, people’s intentions.’*

CPPE.

Providers such as BMJ learning and HEE look at completion rates or turnover, as in the quote below:

*‘When it comes to courses, we commission and continue to do, we have contract with evaluation points throughout the year that looks at turnover, student feedback, attainment in previous years, and we have an annual quality setting process with providers.’*

HEE.

A few stated that no evaluation takes place of the learning events (dentist, Malaysia, Pakistan). When describing the content of the evaluation forms, it is seen that similar questions are asked, covering what went well and what could be completed differently, and in a few cases the intention of how the learning would be applied. 

### 3.6. Opinions on the Best Model for Professional Development Activity

A combination of face-to-face and online was seen to be a good approach, with individuals having individual preferences according to age, learning style, work pattern, or experience:

*‘I think you will find most people do well with a balance, um, and also depends on individuals learning style and what they like best.’*

BMA.

*‘I think both are really valuable. I think online is more accessible if you work shifts.’*

Paramedic.

*‘We do have the very traditional GPs where people want to read and do tests for an hour, and then we have the much younger group that have completely different ways to learn, and they just want to watch a video or something, so we are moving in that direction....’*

BMJ learning.

*‘A lot of people don’t have much flexibility due to hours of work and what they do, so as much as they might want to go along to an evening meeting, where others might feel supported there, they might need to be in their pharmacy stores till late, by which time there is no enthusiasm to go on and do the things that are seen to be less of a requirement, so something a bit more passive might be okay.’*

Pharmacist GB.

Different formats suit different topics, such as skill development needing face-to-face interventions, whereas knowledge updates are suitable to be completed online. This was noted more than once, as seen below:

*‘So, where you are looking for skill development you need to have face-to-face if you are going to assess whether the objectives have been achieved… For others we rely more on online, so, for example, do you need to have face-to-face training for an update on what the changes in flu vaccine are every year, no....’*

Ireland.

*‘I think it depends on the topic you are covering, so, there are some things like, say you want to get emergency hormonal contraceptive pill accredited, the online course is sufficient, followed by, you know, a test at the end. When you want to discuss more emerging themes and stuff like that then I think face-to-face is significant to make those connections and it is more improvement-based initiatives.*

New Zealand 2.

Face-to-face delivery is seen to offer the advantage of being able to share ideas, network, and have hands on experience. Geography and the time of event, along with time needed to attend, were seen as barriers for face-to-face attendance.

*‘I think face-to-face will always have that human element to it, and you will have an opportunity to interact with peers, and learn with them.’*

New Zealand 1.

*‘Distance is a barrier in Australia… because they have, like, rural locations, so they are quite adept to skype and teleconference and running things so you do things remotely… It is a massive place, so to run national and things like that it is often online, teleconference.’*

Australia.

Online learning was a good opportunity for those who work shifts or those who found time to be an issue, as it can be completed in an individual’s own time and online overcomes geographical issues. Younger learners may also prefer this method:

*‘Online is so good because you can just do it when you want. Some of it you can start it and then pause it and go back to it.’*

Dentist.

The cons of online learning were that it is seen as a ‘tick-box’ exercise and not taken as seriously, with learners just being able to click through the material:

*‘Face-to-face is the best, as e-learning, it does not, it is something that is not quite popular as people don’t take it as seriously as such, they just think it is something they can do in their own time, and they are mentally absent during those sessions as well.’*

Pakistan.

As seen above, it was noted that there should not be a ‘one size fits all’ approach as individuals have different preferences. 

## 4. Discussion

It is seen that, although all healthcare professions surveyed in GB are required to undertake CPD, how this is completed varies. Three professions; medicine, nursing, and pharmacy outline revalidation requirements that include CPD. Both pharmacists and dentists have seen changes in requirements in recent years. As echoed in previous international studies [1,5,6], most countries, as represented by the interviewees (9 out of 13), had mandatory CPD/CE requirements for pharmacists in place. Even in those countries where professional development activity requirements were not specified for pharmacists, a culture of wanting to learn and stay up to date was seen. Whilst, for pharmacists in GB, CPD is part of revalidation in line with doctors and nurses, giving assurance of joint emphasis on patient outcomes, revalidation was not mentioned by any of the other pharmacists in the sample population in this study. 

This study shows that most CPD is completed in the professional’s own time, and this could mainly be guided by personal learning needs, as for pharmacists in GB. When looking at the literature, Pool et al. [23] in 2016 saw that for nurses, mandatory courses were useful for complying with requirements, conferences were used for deepening knowledge, and postgraduate education was used to develop careers. Although mandatory to complete CPD for all professions, this study showed that only a few records are checked by the regulators annually. This may impact on the importance professionals place on the entry. When surveyed in 2009 [24], it was seen that 70.5% of nurses had participated in CPD in the past year. In a systematic review of CPD attitudes of pharmacists in GB [2] it was identified that overall, pharmacists understood the benefits of CPD participation; however, there was no overall acceptance and uptake of CPD. In a study carried out by Thompson et al. in 2013 [25], 3 years after mandatory CPD was introduced in Australia, 91% of respondents believed they knew the CPD requirements for renewal of registration. However, registrants could not understand the difference between CPD and CE, with 76% believing they were synonymous. They believed that more guidance on the frameworks available was needed.

This study identified the main providers of training, which included professional bodies, employers, and pharmaceutical companies. These findings are supported by the FIP CPD/CE global report from 2014 [5] that showed from 66 surveyed countries, that 59 (90.6%) used professional associations for pharmacist training. The report showed 83.1% of countries (54/66) used universities as providers, 55.4% (36/66) used employers, 30.8% (20/66) used regulators, and over half of the countries (34/66, 52.3%) used private providers. If mandatory requirements are met, or opportunities are provided to keep up to date, the provider should be the one best placed to deliver content, as previous studies have identified that the facilitator is central to ensuring learning [2,26]; learning that is relevant to practice is seen to support engagement. Previous literature has cited the benefits of employer interventions/learning to support practical experiences. The studies show that if a manager supports their team in learning, this is more likely to have an impact [27,28,29]. Schindel et al. [30] in 2019 showed that peer and manager support is needed in a time of changing practice, and that CPD was successful if it encompassed learning in practice and the workplace. Any CPD event is not truly successful, unless learning has been translated into practice. O’Loan in 2019 [31], when surveying pharmacists in Northern Ireland, noted that professional practice improved more when pharmacists had undertaken structured CPD, and especially where it incorporated workplace learning activities. Feedback from service users supported the demonstration of learning into practice. CPD is becoming more evolved globally as the importance of ongoing education is recognised [32].

Belgium has taken the approach that one provider delivers all the required content on multiple occasions to ensure consistency across the country. The CPPE model in GB is in this manner. By having a centralised approach, this supports quality assurance of the process, and of the providers, which is important to ensure consistent approaches. Ireland and USA utilise accreditation bodies to support assurance. Indeed, in their annual report from 2017 [33], the IIOP stated that the system in place provided quality assurance and ensured patient needs were being met with 95% engaging in the process.

Previous studies have highlighted that mandatory requirement for achieving professional development activity increases participation in events [34]. In this study, where mandatory professional development activity requirements are in place, it is seen that proof of attendance is required in multiple places for verification by the regulator. This places the emphasis on the pharmacist to demonstrate they have participated in the learning. However, it does not prove that application of learning into practice has been achieved. A variety in experience of support for application of learning was seen in this study, with some countries showing a culture of additional tools being provided in most cases. Ireland has a system to ensure that pharmacists can demonstrate their learning in practice. 

This study emphasises the findings of previous studies that there is no ‘one size fits all’ approach for professional development activity, with advantages and disadvantages of online and face-to-face learning events [35]. This echoes a previous study [36] by Driesen et al. in 2007, that showed differing formats to be preferable for different audiences. When looking at age preferences, previous research by Simonds [37] in 2014 showed that with online learning, older students preferred to watch lectures, whereas younger students preferred more interactive learning strategies. A previous study [38] identified that those aged 36–45 had lower preference for face-to-face learning, possibly linking to child-bearing age. The study identified that when considering pharmacists, even though younger pharmacists were open to technology and online learning, they did not want it to replace face-to-face contact completely [38], which was also found by Simonds [37]. 

Multiple previous studies have focused on format preferences, and the pros and cons of each, and this study was no different. Face-to-face interactions allow participation and two-way interaction, as well as the ability to ask questions, along with networking and sharing experiences with peers. Whilst most face-to-face interactions at the time of this study were in person, more face-to-face online sessions are now available. Online independent learning with no instructor is a more flexible option that allows work at the participants own pace and in their own time but limits the ability to engage with an instructor. Blended learning supports the ability to have some face-to-face learning, along with some self-directed study. There are challenges when introducing blended learning, as complex planning, time, and resources are required, but there are multiple opportunities with this approach [39]. A study from pharmacists in the Middle East showed that whilst pharmacists appreciated the ability of online learning to be flexible, the challenges they faced included technology, time management, and learner isolation [40]. This study highlighted that different topics were more suitable for various formats, with the hands-on service-based learning better suiting face-to-face learning and knowledge updates could be completed online. This has been seen in undergraduate education, where grades improved when content was delivered online followed by team-based classroom activities [41]. A study by Jeffries [42] in 2013 emphasised that e-learning can assist learners to keep up to date with new knowledge. 

In this study, time has been cited as a barrier to CPD; both the time needed for attendance and the time of the day. Pharmacists noted time as their biggest barrier to participation in CPD [2]. Time for face-to-face events was the biggest barrier to attendance in female doctors in a previous study [43]. Geography may affect the ability to join face-to-face events, as seen in this study in Chile or Australia, echoed by a previous study in rural Western Australia [44] that showed that journals were the most common source of education, followed by reference books and the internet. Conversely, smaller countries such as Malta, showed in this study that geography supported face-to-face provision. Location of training has been identified as a barrier for attendance at learning events in multiple previous studies [2,26]. In countries where CPD is not commonplace, lack of opportunities or being unaware of the CPD concept could be a barrier, such as that in a 2018 study of pharmacists in Ethiopia [45]. Nurses and paramedics saw more variety in learning times than other professions, with events occurring during the day or during evenings, perhaps due to shift work to allow for participation. Daytime events were seen for safety reasons, such as in Iraq.

Where evaluation forms are used for events, this study showed that similar questions are asked, focusing on the event itself and the positives and negatives. Limited occasions asked for intention of how the learning would be applied. Seeking understanding of intent to apply learning would support pharmacists in CPD, to complete the cycle demonstrating reflection of practice, which is an integral step of the CPD process, and is an opportunity for the future.

In a systematic review of factors affecting global participation in pharmacy professional activities, completed in 2020 [46], the four factors to increase participation were attitudes, access to needs-based education, support, and policy, showing a collective and policy driven approach is important.

The limitations of this study include that only one person from selected professions or countries, apart from New Zealand, was interviewed so experiences will be varied, and as the information provided by the respondents is self-reported opinions given regarding preferences of learning would be personal and not necessarily representative of the whole profession or country. Country variations may not be represented, e.g., in the USA, where there are variations in requirements in different states. In addition, representatives from professional bodies did not represent all professions, and regulators were not approached. In addition, a purposive approach was taken to gain participants with known pharmacists approached, which may have affected the range of countries sampled, and could have potentially added bias to the results. As interviews took place in 2017 and 2018, information may have changed since this study took place. Formats of learning have also changed after the end of this study, due to the COVID-19 global pandemic.

However, the range of professions and countries represented showed the varied practices that exist globally, demonstrating similarities and differences that can be built on in future studies with larger cohorts, and that can give ideas for countries currently with no regulations who may wish to implement changes. 

## 5. Conclusions

It is seen that CPD is commonplace across healthcare practitioners in GB, similar to pharmacists, and professions see the benefit of completing learning. For GB based pharmacists, CPD is now part of revalidation requirements, akin to doctors and nurses, showing alignment of practice for the three largest healthcare professions in GB, supportive of a multi-professional approach to patient care. From the sample interviewed in this study, global revalidation for pharmacists was not mentioned explicitly. Globally for pharmacists, a variety of models of CPE/CE exist to ensure they are up-to-date, and even where mandatory systems are not in place, there is a motivation from pharmacists to participate in learning events. Participation and engagement are dependent on individual preferences and needs. Learnings identified include flexibility in their approach to providing learning, to allow registrants to participate how and when it is suitable for them, and summaries and tools to support application of learning into practice were appreciated. There are still inconsistencies globally about learning expectations, therefore countries and professions should continue to work together to share experiences of processes and learning material to support all pharmacists and other healthcare professionals with keeping up to date with their practice in a way that supports the individual and assures the quality of CPD/CE. Regulations should support this to ensure patient-based practice outcomes. Despite the differences observed, this study showed that commitment to learning is similar in different professions in GB and by pharmacists globally, with similar benefits and challenges. 

## Figures and Tables

**Table 1:** Summary of requirements for CPD/CE for surveyed professions in GB from interviews.

Profession	Mandatory CE or CPD?	How Can Learning Be Achieved	When Learning Takes Place, if Planned Sessions	Providers	Tools to Support Application of Learning	Evaluation of Events
Nurse	CPD	Anything	Evening and during the day. Weekends not common	Royal College of Nursing Educational Institutions	Case studies. Vignettes	Online questionnaire
Dentist	CPD	Verifiable—a certificate is needed Non-verifiable—self-directed learning: e.g., reading brochures, practice meetings	Evenings mostly	British dental journal British Dental Association	Case study approach to learning	None
Doctor	CPD	Portfolio. Demonstration of competencies	Mainly during the day	Deaneries	Online modules. Handouts	Differs by provider
Radiographer	CPD	Mixture of anything	Weekends or lunchtime	Royal college	CPD tool at the end of journal articles giving structured guidance	Self-reflection using CPD portfolios
Paramedic	CPD	Attendance at courses	Evenings and during the day	Hospital Trust Ambulance service	No extra given	Evaluation form
British Medical Association	(Professional body)	Online courses. Attendance at courses in London	2 h in the evening twice monthly	(Are a provider)	Recordings of lectures. Handouts. Relevant BMJ learning article	Survey Monkey^®^
British Medical Journal (BMJ) learning	(Provider)	Online courses	Anytime as online. Hour long modules	(Are a provider)	Framework given to allow self-reflection	Start rates and completion rates. Star ratings
Royal Pharmaceutical Society	(Professional body)	No role in provision	No answer provided as no role in provision	No answer provided as no role in provision	No answer provided as no role in provision	Role in accreditation
Centre for Pharmacy Postgraduate Education (CPPE)	(Provider)	Online e-learning, distance learning, workshops, peer led focal point workshops	Mostly evenings but conferences during the day or at weekends. Lunchtimes	(Are a provider)	Assessments Case studies	Evaluation forms
Health Education England	(Commissioner)	Different things for different professions	No answer provided as a commissioner	Medical schools, Higher Education Institutions	No answer provided as a commissioner	Turnover, student feedback, previous attainment

**Table 2:** Summary of requirements for CPD/CE for pharmacists from surveyed countries.

Country	Mandatory CE or CPD?	How Can Learning Be Achieved	When Learning Takes Place, if Planned Sessions	Providers	Tools to Support Application of Learning	Evaluation of Events
Australia	CPD	No restriction on format, although 50% must involve peers	Monthly local evening seminars. Weekend seminars	Pharmaceutical Society of Australia Society of Hospital Pharmacists	Online assessments	Evaluation form/online survey
Belgium	CE	Attendance of lessons or lectures. Face-to-face or online	Mainly evening. Occasionally weekend	Institute for Permanent Study for pharmacists (IPSA) The Scientific Society of Francophile Pharmacists (SSPF)	10–14 days post even a list of 10 to remember is sent. Follow-up case studies. Online assessments	Evaluation form
Chile	No requirements	Training courses	Ad hoc	The Association of Pharmacists	Case studies. Group working	None
Great Britain	CPD	No restrictions on format. Multiple available	Mainly evening but some daytime sessions during the week or at weekends	The Centre for Postgraduate Pharmacy Education (CPPE). Private providers and professional groups	Assessments Handouts	Evaluation form/online survey
India	No requirements	Training courses	Ad hoc	Ad hoc	Limited	None
Iraq	CPD	All face-to-face	During the day or at weekends	Syndicate of Iraqi pharmacists	Handouts	Evaluation form/online survey
Ireland	CPD	Online and face-to-face	Ad hoc	Irish Institute of Pharmacy (IIOP)	Personal development portfolio	Online survey
Malaysia	CPD	Self-choice	Anytime	Pharmaceutical companies	Limited	None
Malta	No requirements	Face-to-face	After 8 pm. Occasionally on a Sunday	The university. Medicines Authority. Drug companies. Medical school	Case scenarios	Evaluation form
New Zealand	CPD	Mixture of face-to-face and online	Evenings or weekends	New Zealand Pharmaceutical Society. New Zealand Hospital Pharmacists association. Community franchises	Online material. Memory sticks with information. Online assessments. Handouts	Evaluation form/online survey
Pakistan	No requirements	Limited available	Ad hoc	Hospitals may put on for their staff. Nothing for those in industry or community	Limited	None
Philippines	CE	Daytime or evening	Daytime or evening	Philippine Pharmacist Association	No additional	Evaluation form
United States of America	CE or CPD, dependent on state	Conferences, accredited articles and book chapter-based learning, accredited webinars	Daytime	Need to be accredited by Accreditation Council for Pharmacy Education (ACPE) or American Society of Health-System Pharmacists (AHSP). American College of Clinical Pharmacy (ACCP). American Pharmacy Association	Handouts	Evaluation form/online survey

## Data Availability

Data available in a publicly accessible repository that does not issue DOIs. Publicly available datasets were analyzed in this study. This data can be found here: https://eprints.kingston.ac.uk/id/eprint/49419/; accessed on 30 December 2021.

## Appendix A. Interview Questions

1. How do you qualify to register as a healthcare professional?
2. What professional requirements surround the need to complete ongoing education post registration in your profession/country?
3. How does your profession/country currently provide supplementary education post registration?
4. What support, if any, is given to support education post registration?
5. When does learning traditionally take place?
6. Which providers are used for post registration education and training?
7. What do you think is the best model for post registration education and training and why?
8. How is learning recorded and verified?
9. What tools or resources are currently used to help practitioners apply their learning into practice?
10. Are there any tools that are not currently utilised that you feel would be of benefit to support application of learning?
11. How does evaluation of learning events currently occur both at a training event and afterwards?
12. Who carries out the evaluation?
13. Do you have any other comments that you think would be useful?

## Appendix B. COREQ Checklist

	**Item No**	**Guide(s)/Description**	**On Page No**
		Domain 1: Research Team and Reflexivity	
Interviewer/Facilitator	1	Which author/s conducted the interview or focus group? RM	Methods-4
Credentials	2	What were the reseachers credentials? RM-MPharm RK–PhD, MSc, BPharm	Title page
Occupation	3	What was their occupation at the time of the study? RM- senior lecturer RK–Professor	Methods-5
Gender	4	Was the researcher male of female? Females	Methods-4
Experience and Training	5	What experience or training did the researcher have? RM–3 years of prior experience of qualitative research	Methods-5
		Relationship with participants	
Relationship Established	6	Was a relationship established prior to the study commencement? Yes	Methods-4
Participant Knowledge of the Interviewer	7	What did the participants know about the researcher? E.g., personal goals, reasons for doing the research Participants were made aware this was part of a PhD study and emailed an information sheet outlining the aims and objectives of the study	Methods-4
Interviewer Characteristics	8	What characteristics were reported about the interviewer/facilitator? E.g., Bias, assumptions, reasons and interests in the research topic PhD candidate and pharmacist	Methods-4
		Domain 2: Study Design	
		Theoretical Framework	
Methodological Orientation and Theory	9	What methodological orientation was stated to underpin the study? E.g., grounded theory, discourse analysis, ethnography, phenomenology, content analysis Content analysis	Methods-5
		Participant Selection	
Sampling	10	How were the participants selected? E.g., purposive, convenience, consecutive, snowball Convenience	Methods-4
Method of Approach	11	How were the participants approached? E.g., face-to-face, telephone, mail, email Face-to-face and telephone	Methods-4
Sample Size	12	How many participants were approached? 26 were approached; 24 were interviewed	Methods -4 Results-5
Non-participation	13	How many people refused to participate or dropped out? Reasons? No response from pharmacists in 2 countries	Results-2
		Setting	
Setting of Data Collection	14	Where was the data collected? E.g. home, clinic, workplace Workplace for face-to-face	Methods-4
Presence of Non-participants	15	Was anyone else present besides the participants and researchers? No other individuals were present	Methods-5
Description of Sample	16	What are the important characteristic of the sample? E.g., demographic data, date Interviews were conducted between February 2017 and October 2018 24 interviews–5 healthcare professionals in GB, 5 professional body representatives in GB, 14 pharmacists globally	Methods–4 Results-5
		Data Collection	
Interview Guide	17	Were questions, prompts, guides provided by the authors? Was it pilot tested? Semi-structured interviews were used. Questions were provided by the authors. Pilot testing was performed with three pharmacists and one nurse. Face validation recieved	Methods-4/5
Repeat Interviews	18	Were repeat interviews carried out? If yes, how many? No	
Audio/Visual Recording	19	Did the research use audio or visual recording to collect the data? All interviews were audio recorded and transcribed	Methods-4
Field Notes	20	Were field notes made during and/or/after the interview or focus group? No additional notes were made	Methods-4
Duration	21	What was the duraction of the interviews or focus groups? They lasted between 10–15 min	Methods-4
Data Saturation	22	Was data saturation discussed? All those who agreed to participate were included	Methods-4
Transcripts Returned	23	Were transcripts returned to particpants for comments and/pr correction? No	
		Domain 3: Analysis and Findings	
		Data Analysis	
Number of Data Coders	24	How many data coders coded the data? Transcripts were read by both members of the research team (RM, RK)	Methods-5
Description of the Coding Tree	25	Did authors provide a description of the coding tree? Questions were used as codes	Methods-5
Derivation of Themes	26	Were themes identified in advance or derived from the data? Inductive content analysis was used	Methods-5
Software	27	What software, if applicable, was used to manage the data? Data was analysed manually	Methods-5
Participant Checking	28	Did participants provide feedback on the findings? No	
		Reporting	
Questions Presented	29	Were participant quotations presented to illustrate the themes/findings? Was each quotation identified? E.g., participant number Comments were supported with direct quotes from participants who were annonymised by their country or professoinal representation	Methods–5 Results–6–12
Data and Findings Consistent	30	Was there consistency between the data presented and the findings? Yes	
Clarity of Major Themes	31	Were major themes clearly presented in the findings? Yes	
Clarity of Minor Themes	32	Is there a description of diverse cases or discussion of minor themes? No	

## References

[1] Tran D., Tofade T., Thakkar N., Rouse M. (2014). US and international health professions’ requirements for continuing professional development. Am. J. Pharm. Educ..

[2] Donyai P., Herbert R.Z., Denicolo P.M., Alexander A.M. (2011). British pharmacy professionals’ beliefs and participation in continuing professional development: A review of the literature. Int. J. Pharm. Pract..

[3] Mestrovic A., Rouse M.J. (2015). Pillars and foundations of quality for continuing education in pharmacy. Am. J. Pharm. Educ..

[4] Rouse M.J., Trewet C.B., Janke K.K. (2018). Advancing learning to advance pharmacy practice. J. Am. Pharm. Assoc..

[5] International Pharmaceutical Federation (2014). Continuing Professional Development/Continuing Education in Pharmacy: Global Report. https://www.fip.org/file/1407.

[6] Pharmaceutical Society of Ireland (2010). Review of International CPD Models. https://www.thepsi.ie/Libraries/Education/PSI_International_Review_of_CPD_Models.sflb.ashx294.

[7] Bruno A., Bates I., Brock T., Anderson C. (2010). Towards a global competency framework. Am. J. Pharm. Educ..

[8] International Pharmaceutical Federation (2014). Quality Assurance of Pharmacy Education: The FIP Global Framework. http://fip.org/files/fip/PharmacyEducation/Quality_Assurance/QA_Framework_2nd_Edition_online_version.pdf.

[9] Driesen A., Verbeke K., Simoens S., Laekeman G. (2007). International trends in professional development activity for pharmacists. Am. J. Pharm. Educ..

[10] Owen J.A., Skelton J.B., Maine L.L. (2020). Advancing the Adoption of Continuing Professional Development (CPD) in the United States. Pharmacy.

[11] Micallef R., Kayyali R. (2019). A systematic review of models used and preferences for continuing education and continuing professional development of pharmacists. Pharmacy.

[12] Micallef R., Kayyali R. (2020). Why we should create uniform pharmacy education requirements across different countries: A review of current requirements and the need for global regulator input. Curr. Pharm. Teach. Learn..

[13] Office for National Statistics EMP04: Employment by Occupation. https://www.ons.gov.uk/employmentandlabourmarket/peopleinwork/employmentandemployeetypes/datasets/employmentbyoccupationemp04..

[14] The Nursing and Midwifery Council About Us. https://www.nmc.org.uk/about-us/our-role/.

[15] Health and Care Professions Council About Us. http://www.hcpc-uk.org/aboutus/.

[16] National Health Service About Health Education England. https://hee.nhs.uk/about/.

[17] Irvine D. (2005). Patients, professionalism, and revalidation. BMJ.

[18] Gutacker N., Bloor K., Bojke C., Archer J., Walshe K. (2019). Does regulation increase the rate at which doctors leave practice? analysis of routine hospital data in the english NHS following the introduction of medical revalidation. BMC Med..

[19] General Dental Council (2018). Continuing Professional Development. https://www.gdc-uk.org/professionals/cpd.

[20] Karas M., Sheen N.J., North R.V., Ryan B., Bullock A. (2020). Continuing professional development requirements for UK health professionals: A scoping review. BMJ Open.

[21] Elo S., Kyngäs H. (2008). The qualitative content analysis process. J. Adv. Nurs..

[22] Hsieh H., Shannon S. (2005). Three approaches to qualitative content analysis. Qual. Health Res..

[23] Pool I., Poell R., Berings M., Ten Ate O. (2016). Motives and activities for continuing professional development: An exploration of their relationships by integrating literature and interview data. Nurse Educ. Today.

[24] Drey N., Gould D., Allan T. (2009). The relationship between continuing professional education and commitment to nursing. Nurse Educ. Today.

[25] Thompson W., Nissen L.M. (2013). Australian pharmacists’ understanding of their continuing professional development obligations. J. Pharm. Pract. Res..

[26] Marriott J., Duncan G., Namara K.P.M. (2007). Barriers to pharmacist participation in continuing education in Australia. Pharm. Educ..

[27] Lee N. (2011). An evaluation of CPD learning and impact upon positive practice change. Nurse Educ. Today.

[28] Swallow V., Clarke C., Iles S., Harden J. (2006). Work based, professional development activity through professional portfolios: Challenge or reward?. Pharm. Educ..

[29] Dijksterhuis M.G.K., Schuwirth L.W.T., Braat D.D.M., Teunissen P.W., Scheele F. (2013). A qualitative study on trainees and supervisors perceptions of assessment for learning in postgraduate medical education. Med. Teach..

[30] Schindel T.J., Yuksel N., Breault R., Daniels J., Varnhagen S., Hughes C.A. (2019). Pharmacists’ learning needs in the era of expanding scopes of practice: Evolving practices and changing needs. Res. Soc. Admin. Pharm..

[31] O’Loan L. (2019). Continuing professional development (CPD) for pharmacists: Implications for professional practice. Pharm. Educ..

[32] Sherman L.T., Chappell K.B. (2018). Global perspective on continuing professional development. Asia Pac. Sch..

[33] Irish Institute of Pharmacy Annual Report 2017. https://iiop.ie/sites/default/files/AD3058%20IIOP%20Annual%20Report%202017%20HIGH%20RES.pdf.

[34] Austin Z., Marini A., MacLeod Glover N., Tabak D. (2006). Peer-mentoring workshop for continuous professional development. Am. J. Pharm. Educ..

[35] Gould D., Drey N., Berridge E. (2007). Nurses’ experiences of continuing professional development. Nurse Educ. Today.

[36] Driesen A., Airaksinen M., Simoens S., Laekeman G. (2007). What if continuing education became mandatory? Opinions of Belgian community pharmacists. Int. J. Pharm. Pract..

[37] Simonds T.A., Brock B.L. (2014). Relationship between age, experience, and student preference for types of learning activities in online courses. J. Educ..

[38] Micallef R., Kayyali R. (2018). Factors affecting a face to face learning event. Int. J. Pharm. Pract..

[39] Schindel T.J., Hughes C.A., Sadowski C.A. (2013). Blended learning: Reflections on teaching experiences across the pharmacy education continuum. Pharmacy.

[40] Wilbur K., Taylor A.D. (2018). Does a blended learning environment suit advanced practice training for pharmacists in a Middle East setting?. Int. J. Pharm. Pract..

[41] Hughes P.J., Waldrop B., Chang J. (2016). Student perceptions of and performance in a blended foundational drug information course. Curr. Pharm. Teach. Learn..

[42] Jeffries J.A., Jeffries P.R., Hertig J.B., Hultgren K.E. (2013). Embracing pharmacy E-learning: Models of success. Pharmacy.

[43] Ikenwilo D., Skåtun D. (2014). Perceived need and barriers to continuing professional development among doctors. Health Policy.

[44] Clifford R.M. (2011). Post-registration learning trends of community pharmacists. J. Pharm. Pract. Res..

[45] Gelayee D.A., Mekonnen G.B., Birarra M.K. (2018). Involvement of community pharmacists in continuing professional development (CPD): A baseline survey in Gondar, northwest Ethiopia. Glob. Health.

[46] Alhaqan A., Smith F. (2020). A global evidence review of systemic factors influencing participation in pharmacy professional development activities. Res. Soc. Admin. Pharm..

